# Functional landscape of genome-wide postzygotic somatic mutations between monozygotic twins

**DOI:** 10.1093/dnares/dsae028

**Published:** 2024-09-22

**Authors:** Kenichi Yamamoto, Yoko Lee, Tatsuo Masuda, Keiichi Ozono, Yoshinori Iwatani, Mikio Watanabe, Yukinori Okada, Norio Sakai

**Affiliations:** Department of Statistical Genetics, Osaka University Graduate School of Medicine, Suita, Japan; Department of Pediatrics, Osaka University Graduate School of Medicine, Suita, Japan; Laboratory of Children’s health and Genetics, Division of Health Sciences, Osaka University Graduate School of Medicine, Suita, Japan; Laboratory of Statistical Immunology, Immunology Frontier Research Center (WPI-IFReC), Osaka University, Suita, Japan; Center for Twin Research, Osaka University Graduate School of Medicine, Suita, Japan; Center for Twin Research, Osaka University Graduate School of Medicine, Suita, Japan; Laboratory of Child Healthcare and Genetic Science, Division of Health Sciences, Osaka University Graduate School of Medicine, Suita, Japan; Graduate School of Humanities and Sciences, Nara Women’s University, Nara, Japan; Department of Statistical Genetics, Osaka University Graduate School of Medicine, Suita, Japan; Department of Obstetrics and Gynecology, Osaka University Graduate School of Medicine, Suita, Japan; Department of Pediatrics, Osaka University Graduate School of Medicine, Suita, Japan; Center for Promoting Treatment of Intractable Diseases, ISEIKAI International General Hospital, Osaka, Japan; Center for Twin Research, Osaka University Graduate School of Medicine, Suita, Japan; Department of Clinical Laboratory and Biomedical Sciences, Osaka University Graduate School of Medicine, Suita, Japan; Center for Twin Research, Osaka University Graduate School of Medicine, Suita, Japan; Department of Clinical Laboratory and Biomedical Sciences, Osaka University Graduate School of Medicine, Suita, Japan; Department of Statistical Genetics, Osaka University Graduate School of Medicine, Suita, Japan; Laboratory of Statistical Immunology, Immunology Frontier Research Center (WPI-IFReC), Osaka University, Suita, Japan; Laboratory for Systems Genetics, RIKEN Center for Integrative Medical Science, Yokohama, Japan; Department of Genome Informatics, Graduate School of Medicine, the University of Tokyo, Tokyo, Japan; Premium Research Institute for Human Medicine (WPI-PRIMe), Osaka University, Suita, Japan; Department of Pediatrics, Osaka University Graduate School of Medicine, Suita, Japan; Center for Twin Research, Osaka University Graduate School of Medicine, Suita, Japan; Laboratory of Child Healthcare and Genetic Science, Division of Health Sciences, Osaka University Graduate School of Medicine, Suita, Japan; Center for Promoting Treatment of Intractable Diseases, ISEIKAI International General Hospital, Osaka, Japan

**Keywords:** twin research, monozygotic twin, whole-genome sequencing, postzygotic somatic mutation, age-related clock-like mutational signature

## Abstract

Monozygotic (MZ) twins originate from a single fertilized egg, making them genetically identical at the time of conception. However, postzygotic somatic mutations (PZMs) can introduce genetic differences after separation. Although whole-genome sequencing (WGS) sheds light on somatic mutations in cancer genomics, its application in genomic studies of MZ twins remains limited. In this study, we investigate PZMs in 30 healthy MZ twin pairs from the Osaka University Center for Twin Research using WGS (average depth = 23.8) and a robust germline-calling algorithm. We find high genotype concordance rates (exceeding 99%) in MZ twins. We observe an enrichment of PZMs with variant allele frequency around 0.5 in twins with highly concordant genotypes. These PZMs accumulate more frequently in non-coding regions compared with protein-coding regions, which could potentially influence gene expression. No significant association is observed between the number of PZMs and age or sex. Direct sequencing confirms a missense mutation in the *ANKRD35* gene among the PZMs. By applying a genome-wide mutational signature pattern technique, we detect an age-related clock-like signature in these early postzygotic somatic mutations in MZ twins. Our study provides insights that contribute to a deeper understanding of genetic variation in MZ twins.

## Introduction

A classical twin study is based on the premise that monozygotic (MZ) twins are genetically identical owing to their origin from a single fertilized egg. This assumption has enabled classical twin studies to quantify genetic or environmental contributions to complex traits. In phenotypically discordant MZ twin pairs, environmental factors may contribute to this difference.^1^ Recent advances in high-throughput sequencing technologies have revealed genome-wide genetic differences between MZ twins.^2–4^ Notably, these differences have been shown to contribute to the phenotypes in phenotypically discordant MZ twins with neuropsychiatric^5–7^and autoimmune diseases.^8,9^

The genetic differences within MZ twin pairs are postzygotic somatic mutations (PZMs). PZMs are DNA changes occurring in body cells after fertilization caused by errors during DNA replication or environmental factors such as smoking, or ultraviolet radiation, and accumulate over time. Reference cancer genomics studies have established catalogs of somatic mutational signatures based on different mutagens.^10,11^ Similarly, somatic mutations in normal cells or tissues have been shown to be specific to certain mutagens and accumulate with age.^12–14^ Although these studies shed light on mutational mechanisms in normal cells or tissues, background heterogeneity can hinder the evaluation of PZMs. This requires large-scale, unbiased participant groups for high-resolution analysis. Twin studies, with the advantage of a nearly identical early life background in MZ twins, offer a unique opportunity to investigate the mutational process with less confounding factors. Recent large-scale studies using whole-genome sequencing (WGS) have elucidated the characteristics, timing, and intrafamilial accumulation of genetic differences between MZ twins.^4,15^ However, studies on the genetic differences in MZ twins remain limited, and further evaluations are needed to elucidate the PZMs in a multidimensional aspect.^16^

To elucidate the postzygotic somatic mutational differences between MZ twins on a genome-wide scale, we conducted WGS of 30 healthy adult MZ twin pairs registered at the Osaka University Center for Twin Research, a valuable multidisciplinary resource in Japan.^17^ We detected highly confident postzygotic somatic mutations using a robust variant calling pipeline and identified the functional landscape of these mutations through mutational signature analysis. This study provides an important opportunity to advance the understanding of twin genomics.

## Materials and methods

### Study participants

We enrolled 30 adult MZ twin pairs for WGS analysis (*n* = 60). All the participants were registered at the Osaka University Center for Twin Research, which maintains a volunteer-based twin registry and is open to new enrolments at all times from any region of Japan. Twins of all ages were eligible for participation. As of 1 June 2019, a cumulative total of over 450 twin pairs have been registered.^17^ The zygosity of all participants was confirmed using short tandem repeats. Details of enrollment have been described previously.^18^ Written informed consent was obtained from all the participants. This study was approved by the Research Ethics Committee of Osaka University (approval no. 696).

### WGS analysis

Genomic DNA was extracted from the peripheral blood of participants. To assess the genetic differences between MZ twins on a genome-wide scale, WGS was performed on a HiSeq 2,500 (Illumina) using a 259-base paired-end protocol at the Tohoku Medical Megabank Organization.^19^ The quality of the sequenced reads was checked using the FastQC software. Low-quality reads (Phred quality score < 20) and sequenced adapters were trimmed using Trimmomatic (version 0.36). To detect PZMs with high variant allele frequency, a variant call workflow was performed according to the Genome Analysis Toolkit (GATK) best practice guidelines used to detect germline variants.^20,21^ Trimmed reads were aligned to the human reference genome (hg38) using Burrows-Wheel Aligner with maximal exact matches (MEM) option (version 0.7.15). Duplicate reads were removed using the Picard software (version 2.0.9). Base quality score recalibration was performed using GATK software (version 3.7.0). Individual variants were called using the GATK HaplotypeCaller in the GVCF mode. Participants were jointly genotyped using the GATK CombineGVCF and GenotypeGVCF tools for each autosomal chromosome. Genotypes within the autosomal region satisfying the following criteria were filtered out based on our previous workflow^22^: (i) DP < 5, (ii) GQ < 20, or (iii) DP > 60 and GQ < 95. Variants with low call rates (< 0.90) were also excluded. Then, the variant call accuracy was estimated using Variant Quality Score Recalibration in GATK. To obtain the highly confident variants, we removed the variants located in the following regions: (i) low-complexity regions of hg38 lifted over from GRCh37 defined by mdust^23^ (downloaded from ftp://ftp.1000genomes.ebi.ac.uk/vol1/ftp/release/20130502/supporting/low_complexity_regions/hs37d5-LCRs.20140224.bed.gz), (ii) telomere, centromere, heterochromatin, and the short arm of acrocentric chromosomes in chr13,14,15,21, and 22 defined by CytoBand, and (iii) low accessible genomic regions known by short-read WGS of 1,000 Genomes Project (downloaded from ftp.1000genomes.ebi.ac.uk/vol1/ftp/data_collections/1000_genomes_project/working/20160622_genome_mask_GRCh38/StrictMask/20160622.allChr.mask.bed). In this study, we restricted the analysis to single nucleotide variants (SNVs) in autosomes for robustness. Additionally, we calculated variants allele frequency (VAF) for each variant using GATK AllelicBalanceBySample using bam files, and filtered out regions with low VAFs (< 10%) and low allelic count (< 2) for each individual’s variant call format (VCF) file to minimize the sequencing noises.^13,24^

### Genotype concordance evaluation

We robustly evaluated genotype concordance for SNVs within MZ twins using GenotypeConcordance (Picard). For each MZ pair, we created a 3 × 3 genotype contingency matrix, in which all combinations of the 3 SNV genotypes of the 2 WGS datasets were assigned (reference/reference [Ref/Ref], reference/alternative [Ref/Alt], and alternative/alternative [Alt/Alt]).^25^ Between MZ twins, following 6 genotype pairs are existing; Ref/Ref~Ref/Ref, Ref/Ref~Ref/Alt, Ref/Ref~Alt/Alt, Ref/Alt~Ref/Alt, Ref/Alt~Alt/Alt, and Alt/Alt~Alt/Alt. Because we could not count the number of Ref/Ref-Ref/Ref variants, the genotype concordance rate within the MZ pair was calculated using the following formula:


$$\begin{array}{l} \frac{\begin{array}{c} sum\;of\;the\;number\;of\;variants\;\;(Ref/Alt∼Ref/Alt\;and\;Alt/Alt∼Alt/Alt) \end{array}}{\begin{array}{c} sum\;of\;the\;number\;of\;variants\;\;(Ref/Ref∼Ref/Alt,Ref/Ref∼Alt/Alt,Ref/Alt\;∼Ref/Alt,Ref/Alt\;∼Alt/Alt,\;and\;Alt/Alt∼Alt/Alt) \end{array}}. \end{array}$$

### Detection and annotation of postzygotic somatic mutations

We curated discordant variants, referred to as Ref/Ref-Ref/Alt or Ref/Alt-Alt/Alt, between the MZ twins. For these variants, we performed functional annotation using ANNOVAR (version 2017Jun08). Gene-based annotation was performed using the National Center for Biotechnology Information (NCBI) reference sequence (RefSeq) database. To minimize germline variant contamination and obtain highly confident PZMs, we filtered out the variants registered in the public germline variant databases (eg the Genome Aggregation Database [gnomAD], 1KG, the 3.5kJPN of the Tohoku Medical Megabank, and dbSNP).^13,24^ We confirmed the selected nonsynonymous postzygotic somatic mutations using Sanger sequencing and Integrated Genomics Viewer. Associations between the number of postzygotic somatic mutations and factors such as age^2^ and sex were analysed via multivariate linear regression using the lm() function implemented in R statistical software (version 4.1.2).

### Mutational signature analysis for postzygotic somatic mutations

We conducted a mutational signature analysis for PZMs using SomaticSignatures implemented in *R*.^26^ All mutations deconvolved into the following 6 base substitutions owing to the difficulty of DNA strand differentiation: C > A, C > G, C > T, T > A, T > C, and T > G. Additionally, incorporating the flanking 3ʹ and 5ʹ bases to each substitution, 96 possible sequence motifs are generated. By extracting mutational information, such as chromosomal number, position (hg38), reference allele, and alternative allele, we aggregated 96 mutational motifs for each MZ twin pair. To capture the background mutagenesis of the somatic mutations, we downloaded Mutational Signature v2, which contains the motif frequency data of the representative 1 to 30 mutational signatures derived from unique mutagens from the Catalogue of Somatic Mutations in Cancer (COSMIC) site. For 7 MZ twin pairs with a genotype concordance rate of less than 99%, we created a motif of sequencing artefact patterns, considering the possibility of including sequencing artefacts. We evaluated the similarity of the patterns of PZMs to the 30 mutational signatures using as follows. First, we calculated the log-likelihood value for each MZ twin pair using the formula:


$$\begin{array}{l} logL\left(x\right)=\log n!-\underset{k=1}{\overset{K}{\sum }}\;\log x_{k}!+\overset{K}{\underset{k=1}{\sum }}x_{k}\log p_{k} \end{array}$$

where $x_{k}$ is the observed number of motif k, $\underset{k=1}{\overset{K}{\sum }}\;x_{k}=n$, $p_{k}$ is the frequency of motif k, $\underset{k=1}{\overset{K}{\sum }}\;p_{k}=1$. Second, we complemented the zeros of each of the 30 mutational signatures with half of the minimum value to calculate the log-likelihood. Third, the log-likelihood values for each MZ twin pair were corrected by subtracting the value of the original artefact pattern constructed from the 7 low concordant twins and dividing it by the number of PZMs mutations. Finally, we defined the signature with the highest value as a similar signature.

## Results

### Study participants

We examined 30 healthy adult MZ twins (60 individuals) of Japanese ancestry with confirmed zygosity recruited from the Osaka University Twin Research Center.^17^ The average age of the 30 MZ twins in this study was 49.8 years (ranging from 21 to 82 years old). The sex of each MZ twin pair was concordant with a female sex ratio of 50%. None of the participants had congenital anomalies or diseases. We excluded triplets or more to detect differences between MZ twins. The participants’ information is listed in Table 1.

**Table 1.** The characteristics of participants and WGS results.

Twin pair	Age (y)	Sex	Mean depth of coverage (Twin1/Twin2)	Genotype concordance rate	The number of discordant variants	Exonic region variants	Splice site variants	Nonsynonymous variants	Stop gain/loss variants
1	24	M	25.0/24.5	99.7	52	0	0	0	0
2	37	M	25.1/25.2	99.7	63	4	0	3	0
3	82	M	25.5/24.8	99.7	77	2	0	0	0
4	59	M	24.5/24.9	99.7	163	1	0	1	0
5	79	F	25.6/24.8	99.7	70	1	1	1	0
6	77	F	24.9/24.7	99.6	91	1	0	0	0
7	56	M	24.7/24.6	99.6	49	0	0	0	0
8	46	F	25.2/24.5	99.6	68	1	0	1	0
9	54	M	25.0/24.0	99.6	55	0	0	0	0
10	30	M	24.0/24.8	99.6	55	1	0	0	0
11	48	F	24.6/24.4	99.6	67	0	0	0	0
12	67	F	24.2/23.1	99.6	51	0	0	0	0
13	71	M	23.9/24.1	99.6	164	4	0	3	0
14	27	M	24.0/24.4	99.5	71	1	0	1	0
15	32	M	23.8/23.3	99.5	75	0	0	0	0
16	65	M	23.7/24.7	99.5	112	1	1	1	0
17	41	F	23.5/23.4	99.5	86	2	0	1	0
18	36	F	23.9/23.7	99.5	84	1	0	1	1
19	50	M	23.6/23.8	99.5	82	2	0	1	0
20	29	F	24.6/23.9	99.4	73	2	0	2	0
21	40	M	23.4/22.8	99.3	123	0	0	0	0
22	32	F	21.6/23.9	99.3	114	1	0	1	0
23	37	F	22.5/21.9	99.2	140	2	0	2	0
24	24	F	23.7/23.2	98.9	189	0	0	0	0
25	51	F	22.8/22.7	98.9	314	1	0	1	0
26	21	F	23.9/22.4	98.2	633	1	0	1	0
27	62	F	24.6/19.9	98.1	302	0	0	0	0
28	76	M	21.6/22.4	97.7	951	1	0	0	0
29	66	M	23.5/23.4	96.9	618	2	0	2	0
30	74	F	22.3/22.4	96.5	1811	2	0	1	0

WGS; whole-genome sequencing, M; Male, F; Female. The red line represents the threshold of genotype concordance rate.

### Whole-genome analysis and genotype concordance/discordance between MZ twins

We performed a WGS analysis of the 60 individuals to capture the postzygotic somatic mutational landscape in the genomes of the MZ twins. Low-quality reads were excluded based on the initial quality check of the sequenced reads to retain the accuracy of the detected variants. The mean depth of WGS data was 23.8×. We focussed on the postzygotic somatic differences, especially those originating after separation, in terms of accuracy and phenotypic impact. To assess the highly confident PZMs, we applied a general method to call germline variants.^22^ After stringent variant filtering, we targeted approximately 2.2 Gb, obtaining around 6.9 million variants (TiTv ratio = 2.1 by bcftools version 1.4.1) with an average of 2.5 million variants per sample. For each MZ twin pair, we calculated genotype concordance using a 3 × 3 genotype contingency table. The average genotype concordance rate was 99.2%, with a median value of 99.5% (ranging from a minimum of 96.7% to a maximum of 99.7%; Supplementary Fig. 1). Our genotype concordance rate was consistent with that reported in previous studies.^6,9,27^

**Fig. 1.**
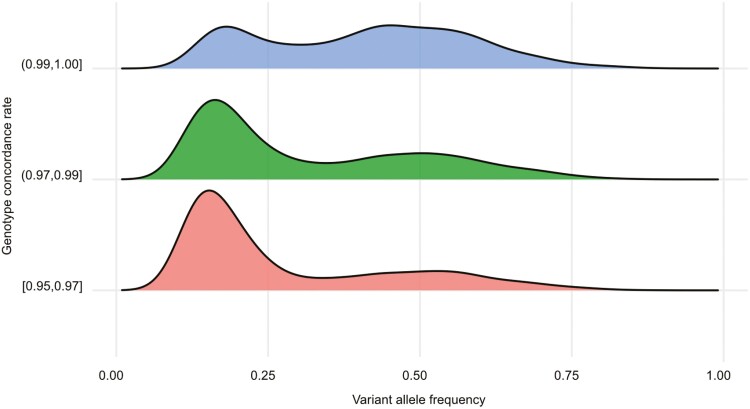
Distributions of variant allele frequency of postzygotic somatic mutations between MZ twins. The distributions of variant allele frequency of postzygotic somatic mutations stratified by genotype concordance rate.

After filtering the publicly known germline variants to minimize contamination and potential artefacts, we obtained a set of PZMs. While the precise timing of these mutations cannot be definitively determined from this data, they represent somatic alterations that arose after fertilization. The average number of postzygotic somatic mutations in the 30 MZ twin pairs was 227. When we restricted the 23 MZ twins to those with a high concordance rate (cut-off of 99%), the average number of PZMs (86, range: 49–164; Table 1) was similar to previous report.^4^ The distribution of VAFs for PZMs revealed a higher frequency of mutations with VAF around 0.5 in high genotype concordant twins compared with other twins (Fig. 1). In contrast, the frequency of somatic mutations with low VAF increased with a decrease in genotype concordance rate. VAF and genotype concordance rate were positively associated even after the adjustment for age and sex (Table 2). Notably, a separate analysis showed a negative association between age and VAF (Beta = −2.2 × 10^−3^, Standard error = 1.1 × 10^−4^, *P* = 1.1 × 10^−88^ in univariable regression, Supplementary Fig. 2). The stratification of the VAF distribution based on the genotype concordance rates and functional regions showed that the VAF distributions were almost concordant in exonic region regardless of genotype concordance. However, low concordant twins maintained a higher frequency of low VAF somatic mutations in non-exonic region (Supplementary Fig. 3). These results indicate the potential noises in low VAF mutations.

**Table 2.** Multivariable linear regression results of variant allele frequency of postzygotic somatic mutations between 30 MZ twins.

Factors	Beta	Standard error	T value	P
Concordance rate	3.3	0.22	14.9	1.2 × 10^−49^
Age	−1.2 × 10^−3^	1.4 × 10^−4^	−8.5	2.4 × 10^−17^
Sex	−1.6 × 10^−2^	4.8 × 10^−3^	−3.3	9.6 × 10^−4^

### Functional characteristics of the postzygotic somatic mutations

**Fig. 2.**
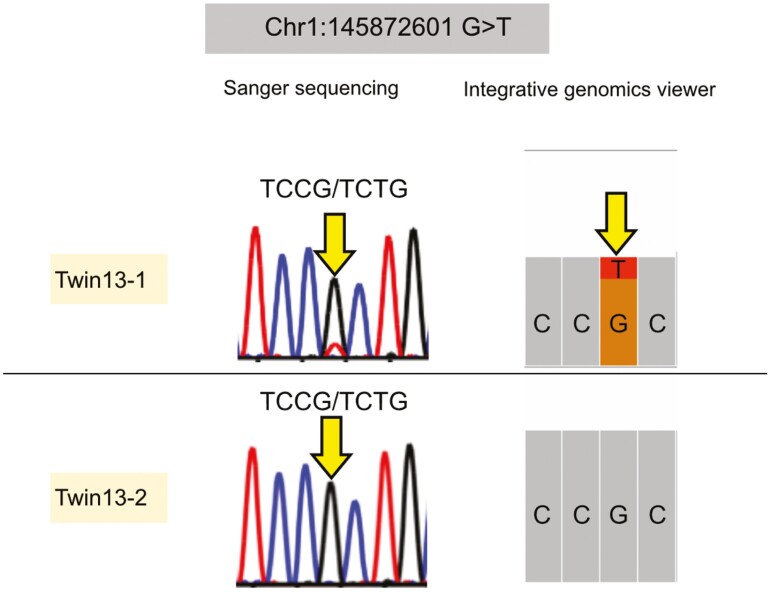
Validation of a postzygotic somatic mutation between MZ twin. We confirmed a postzygotic somatic mutation in the MZ twin using Sanger sequencing and Integrative Genomics Viewer. Among several protein altering variants, a heterozygous missense mutation, *ANKRD35* c.2168C > A (p.Ala723Gle), was validated by Sanger sequencing.

We conducted functional evaluations to determine the genetic differences between the MZ twins. We observed a lower number of PZMs in exonic regions compared with the other regions not related to gene function (*P* = 2.1 × 10^−11^ in Wilcox rank sum test). In the 23 high-genotype concordant twins, the tendency was maintained (*P* = 5.0 × 10^−9^ in Wilcox rank sum test). This finding is consistent with the lower proportion of exonic DNA in the genome. Among some missense mutations, we confirmed 1 heterozygous missense mutation, c.2168C > A (p.Ala723Gle), in *ANKRD35* (NM_144698.5) via Sanger sequencing (Fig. 2). While the mutation and *ANKRD35* gene have not been reported to be associated with any Mendelian disease, an association with granulocyte counts was detected using genome-wide association analysis.^28^ For the other mutations, we could not validate PZMs using Sanger sequencing (Supplementary Table 1). Next, we investigated the association of postzygotic somatic mutations with age and sex. While somatic mutations accumulate with age, we hypothesized that early postzygotic somatic mutations after separation were not influenced by age or sex. According to this hypothesis, the number of PZMs in MZ twins is independent from ages after the adjustment for sex (Beta = 6.1, Standard error = 3.5, *P* = 0.095).

### Signature of the postzygotic somatic mutations

**Fig. 3.**
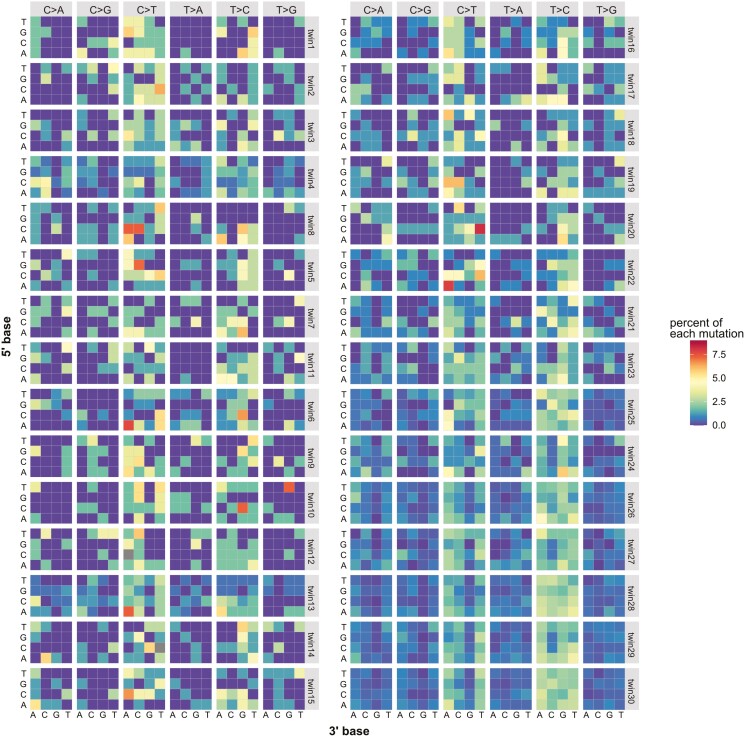
Mutational patterns of postzygotic somatic mutations between MZ twins. Heatmap representing the percentage of each mutational pattern in the 30 MZ twin pairs. The x-axis indicates 3ʹ flanking base and y-axis indicates 5ʹ flanking base. The percentage was calculated using SomaticSignature with normalization.

Somatic mutations occur due to multiple mutational process. Although different exogenous or endogenous aetiologies, such as smoking, ultraviolet radiation, and DNA repair, have been determined based on mutational patterns in cancer genomics,^11^ the mutational process of the genetic differences between MZ twins remains elusive. Thus, we analysed the mutational signature using genome-wide PZMs between MZ twins. The assessment of the mutational type of single-base substitutions revealed the predominance of C > T and T > C substitutions among the 6 substitution types: C > A, C > G, C > T, T > A, T > C, and T > G (Fig. 3).

**Fig. 4.**
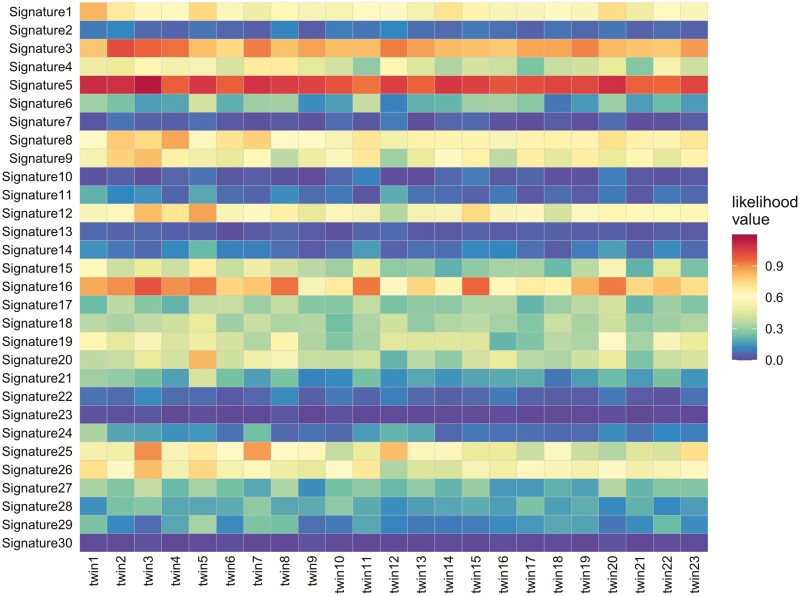
Heatmap showing the similarity of mutational patterns to 30 mutational signatures for 23 MZ twin pairs. The similarity of the mutational signature was evaluated by likelihood value correction using the sequencing artefact pattern and number of variants. The x-axis represents the 23 MZ twin pairs with a high genotypic concordance rate. The y-axis represents the mutational signatures.

Next, we evaluated the mutational pattern constructed from 96 mutational motifs with the base information from the 5ʹ and 3ʹ adjacent nucleotide. Since 7 MZ twins with low genotype concordance rates exhibited the mutations with lower VAFs and a T > C pattern, we considered that these twins might contain more sequence noises than the other twins (Fig. 1, Table 1, and Supplementary Fig. 4). To address this potential bias, we removed the mutational patterns observed in low concordant twins from the analysis of the other twins. This approach aimed to establish a robust reference for genuine somatic mutations independent from the potential artefacts observed in the discordant samples with lower concordance rates. For the remaining 23 MZ twin pairs with high genotype concordance rates, we compared with mutational patterns with 30 well-known mutational signatures from COSMIC. Based on the likelihood values adjusted for the pattern of sequence-related artefacts, we observed an affinity of the postzygotic somatic mutations between MZ twins to Signature 5 (Fig. 4), which is characterized by a T > C substitution and an age-related mutational pattern.^29^

## Discussion

This study aimed to determine the functional landscape of early postzygotic somatic mutations in MZ twins. By analysing the WGS data from 30 healthy adult MZ twin pairs, we identified 3 key features: (i) high genotype concordance rates between MZ twins; (ii) selection pressure of the mutational burden of postzygotic somatic mutations in non-exonic regions compared to exonic regions; and (iii) mutational signature resembling the mutational signature 5, which is known as an age-related clock-like signature.

In this study, we focussed on postzygotic somatic mutations that arise after zygote splitting in MZ twins. Although somatic mutations can occur throughout the lifespan, the phenotypic impact of those that occur later in life is less clear because of their localized nature and low variant allele frequency. In addition, mutations with low variant frequencies have the potential to result in false positives. In contrast, early postzygotic somatic mutations spread to almost all cells in the body and have sufficient variant allele frequencies to impact the phenotype. Thus, instead of employing dedicated somatic mutation calling methods, we applied the general variant calling method to detect germline variants to robustly determine the highly confident postzygotic somatic mutations.^22^ Additionally, we applied strict filtering methods to minimize artefacts originating from the sequencing processes. Briefly, we removed low-quality reads along with sequencing base quality, regions prone to errors (eg repetitive regions, centromeres, etc.), and low variant allele frequency regions. We obtained high genotype concordance rates between MZ twins, similar to the results of a previous study.^4^ However, 7 pairs of the 30 MZ twin pairs had genotype concordance rates under 99%. We considered that the datasets from these MZ twins included sequencing artefacts that potentially differed from the previously reported C > A mutational patterns derived from the sample preparation step.^30^ We conservatively removed these pairs using a mutational signature analysis. Further technical innovations are required to maintain a sufficient sample size.

We defined postzygotic somatic mutations as the genotype pairs Ref/Ref-Ref/Alt or Ref/Ref-Alt/Alt, excluding known germline variants registered in global and ancestral population-specific databases. While this approach is commonly used in cancer genomics, excessively filtering out the public polymorphic sites might lead to increased false-negative rates.^31^ This highlights the need for developing alternative approaches that balance the trade-off between minimizing germline contamination and maximizing the capture rate of true somatic mutations.

The VAF distribution showed a high frequency of PZMs with VAFs around 0.5 in high genotype concordant twins (≥ 0.99). In the origin of PZMs, PZMs with VAFs around 0.5 possibly originated at the early cell division stage, especially the 2-cell stage, while PZMs with VAFs around 0.25 might have been generated at the 4-cell stage. Although our study suggests the potential correlation between VAFs and the timing of cell division, as proposed in the previous study,^4^ we did not have the information about chorionic and amnionic status in our twins. In contrast, the low VAF somatic mutations might be recent mutations in blood cells. To reveal these considerations, further studies using chorionicity, younger twins, and other tissues are warranted.

In mutation rate, the number of PZMs in our study was 86.3 ± 34.1 (mean ± sd) within the 23 high genotype concordant pairs. Even when focussing on the PZMs with VAF from 0.4 to 0.6, considered to originate from early embryonic cell divisions based on VAF distribution (37.7 ± 12.8), the number was slightly higher compared to the expected germline mutation rate. However, our results fall within the range reported in the previous studies,^2,4,15^ and are similar to the numbers of PZMs occurring in the pre-primordial germ cell.^4^ Indeed, we observed no correlation between the number of PZMs and age as previously reported.^15^ The previous studies, not limited to twins, have reported the high mutation rates in the early developmental stage of human and mouse tissues.^32–34^ These findings suggest that the timing of the detected PZMs could be in the pre-PGC stage. However, in some MZ twin pairs, we observed an increase in the number of postzygotic mutations with age. Since our samples were limited to blood cells, this finding might be influenced by clonal haematopoiesis as reported previously.^35,36^ To obtain a more precise estimate of the number of PZMs occurring in this stage, further investigation using other tissues (eg buccal swabs, fibroblasts), additional families, and younger participants will be warranted.^37^ From a functional perspective, we confirmed 1 missense PZM in *ANKRD35* via Sanger sequencing; however, its phenotypic impact on the MZ twins remains unknown. Close observations of the MZ twins are warranted. However, we were unable to confirm all of the missense postzygotic somatic mutations using Sanger sequencing. Considering the limitations of Sanger sequencing for mosaic mutations, deep-coverage targeted sequencing is required.^38,39^

Lastly, genome-wide postzygotic somatic mutations were similar to the mutational signature 5. This mutational pattern has been observed in various cell types, including haematopoietic progenitor stem cells and preneoplastic cells with de novo germline mutations and somatic mutations.^29,40^ A recent multi-tissue study of the same individuals revealed a pattern of Signature 5 under somatic mutations in all cells.^33^ While both Signatures 1 and 5 are age-related, we did not detect a strong signal for Signature 1 in PZMs in the MZ twins. Signature 1 arises from the spontaneous deamination of 5-methylcytosine. Meanwhile, although the detailed mechanism of Signature 5 is still unknown, the involvement of *NOTCH2* gene mutations is suggested.^41^ The association between NOTCH signalling and cell proliferation might explain the predominance of Signature 5 to Signature 1 in PZMs within MZ twins. However, no mutations in *NOTCH2* gene were observed in the present study. Furthermore, we observed a relatively high likelihood value for Signatures 3 and 16. These signals were not associated with age, and further investigation of their relevance is needed.

Nonetheless, this study has some limitations. To reduce the ambiguity caused by somatic indels and structural variants, this study focussed only on SNVs between the MZ twins. Further advancements in long-read sequencing technologies hold promise for providing more definitive results regarding the structural mutations in MZ twins. Additionally, investigating various cell types and tissues with larger sample sizes could provide a more comprehensive understanding of PZMs in MZ twins.

Our findings suggest that postzygotic somatic mutations accumulate independently of age and sex and exhibit a mutational signature similar to age-related mutagenesis between MZ twins. Further research should explore the functional consequences of these mutations and their potential contributions to the differences in twins.

## Supplementary Material

dsae028_suppl_Supplementary_Figures_S1-S5_Table_S1

## Data Availability

FastQC, https://www.bioinformatics.babraham.ac.uk/projects/fastqc/; Trimmomatic, http://www.usadellab.org/cms/?page=trimmomatic; BWA, https://github.com/lh3/bwa; Picard, https://broadinstitute.github.io/picard/; GATK, https://gatk.broadinstitute.org/hc/en-us; Annovar, https://annovar.openbioinformatics.org/en/latest/; bcftools, https://samtools.github.io/bcftools/; Mutational Signature v2, https://cancer.sanger.ac.uk/cosmic/signatures_v2.tt; 3.5K JPN, https://jmorp.megabank.tohoku.ac.jp/201905/; COSMIC, https://cancer.sanger.ac.uk/signatures/; GWAS catalog, https://www.ebi.ac.uk/gwas/; and R, https://www.r-project.org/.
