# Supplementary material for: Functional landscape of genome-wide postzygotic somatic mutations between monozygotic twins
Source: DNA Res. 2024 Sep 22;31(5):dsae028. doi: 10.1093/dnares/dsae028 (PMC11472055; doi:10.1093/dnares/dsae028)
Supplement: dsae028_suppl_Supplementary_Figures_S1-S5_Table_S1 [file dsae028_suppl_supplementary_figures_s1-s5_table_s1.docx]

**Supplementary information**


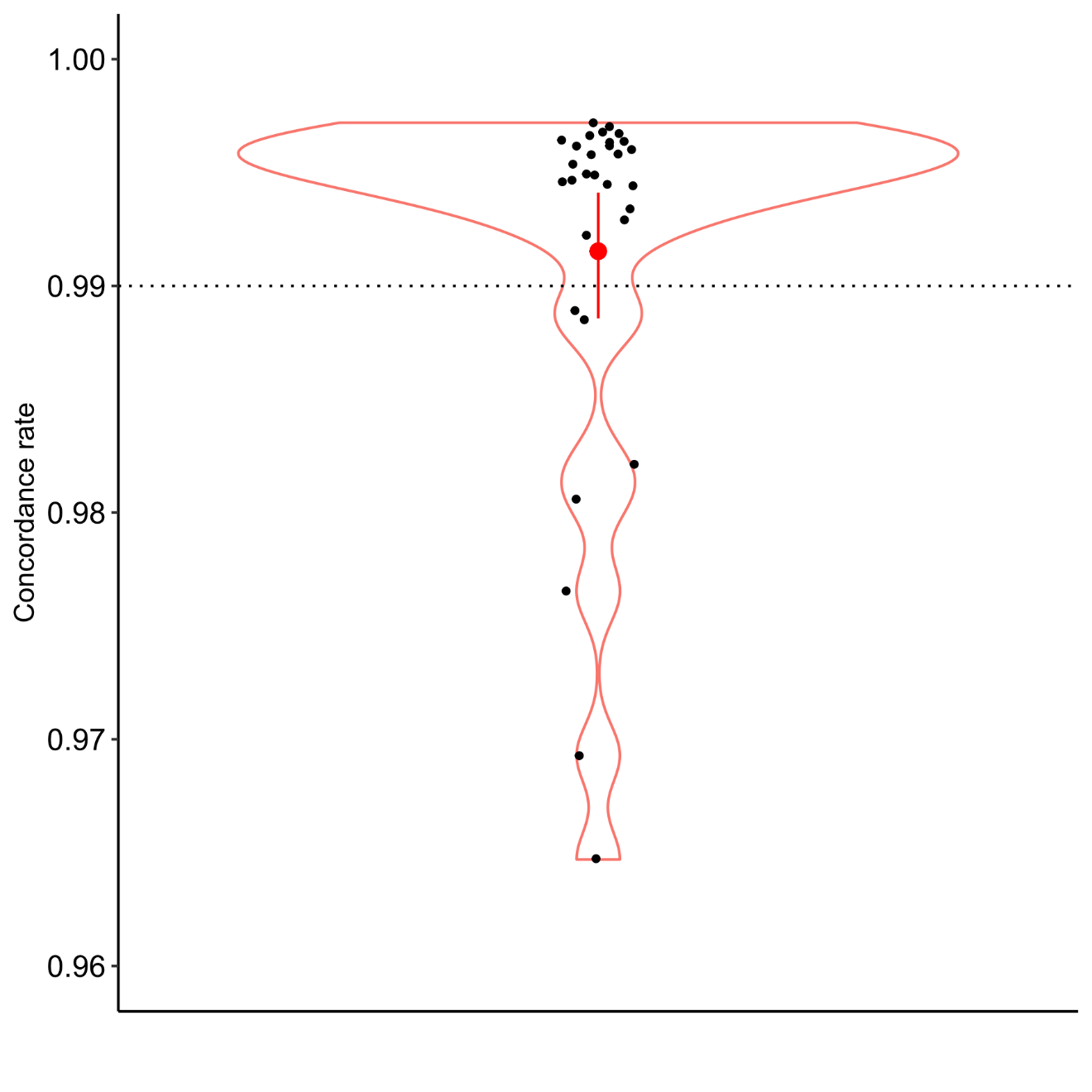


**Supplementary Fig. 1. The distribution of the genotype concordance rate across 30 MZ twins.**

The violin plot of the genotype concordance rates of 30 MZ twin pairs. Each genotype concordance rate was calculated from the number of Ref-Ref/Ref-Alt or Alt-Alt/Ref-Alt variants. The red circle represents the mean and the vertical line is standard deviation. Each dot indicates the each MZ twin pair. The dotted line represents the threshold of 99%.


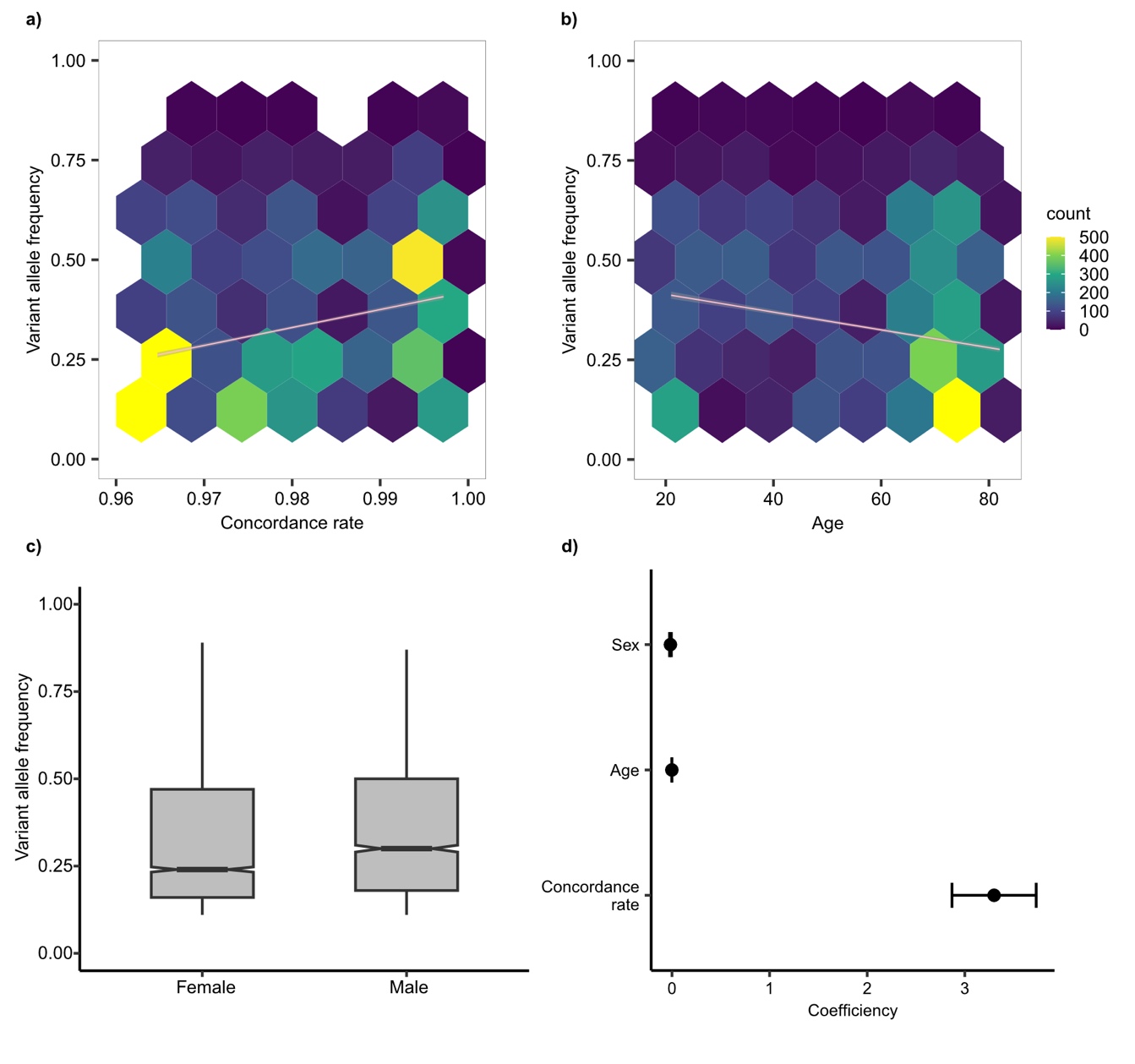


**Supplementary Fig. 2. Associations between variant allele frequency of postzygotic somatic mutations and other factors.**

a) The distribution of variant allele frequencies (VAFs) and genotype concordance rates. b) The distribution of VAFs and ages. The line represents the linear regression. **c)** The boxplots of the distribution of VAFs by sex. The boxes denote the interquartile range (IQR) and the median is shown as notch and black horizontal bars; whiskers extend to 1.5 times the IQR. d) Forest plots represent the effect size of each factor on VAFs. Error bars indicate 95% confidence intervals.


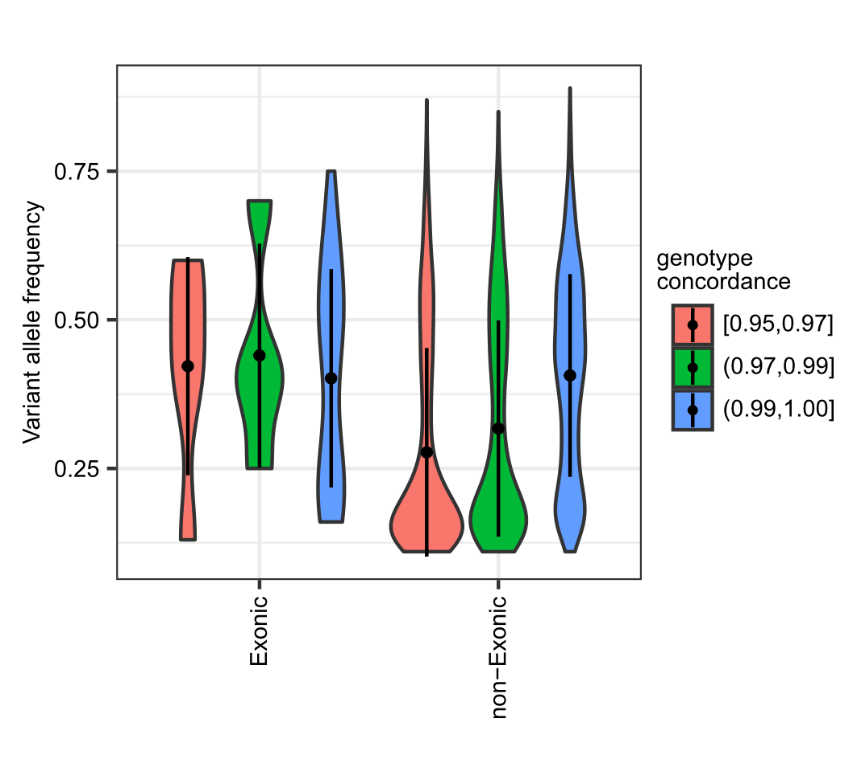


**Supplementary Fig. 3. Distributions of variant allele frequency of postzygotic somatic mutations based on functional regions.**

The distributions of VAF of postzygotic somatic mutations stratified by genotype concordance rates and functional regions (exonic or non-exonic). Violin plots depict the distribution, with dots representing means and vertical lines indicating standard deviations.


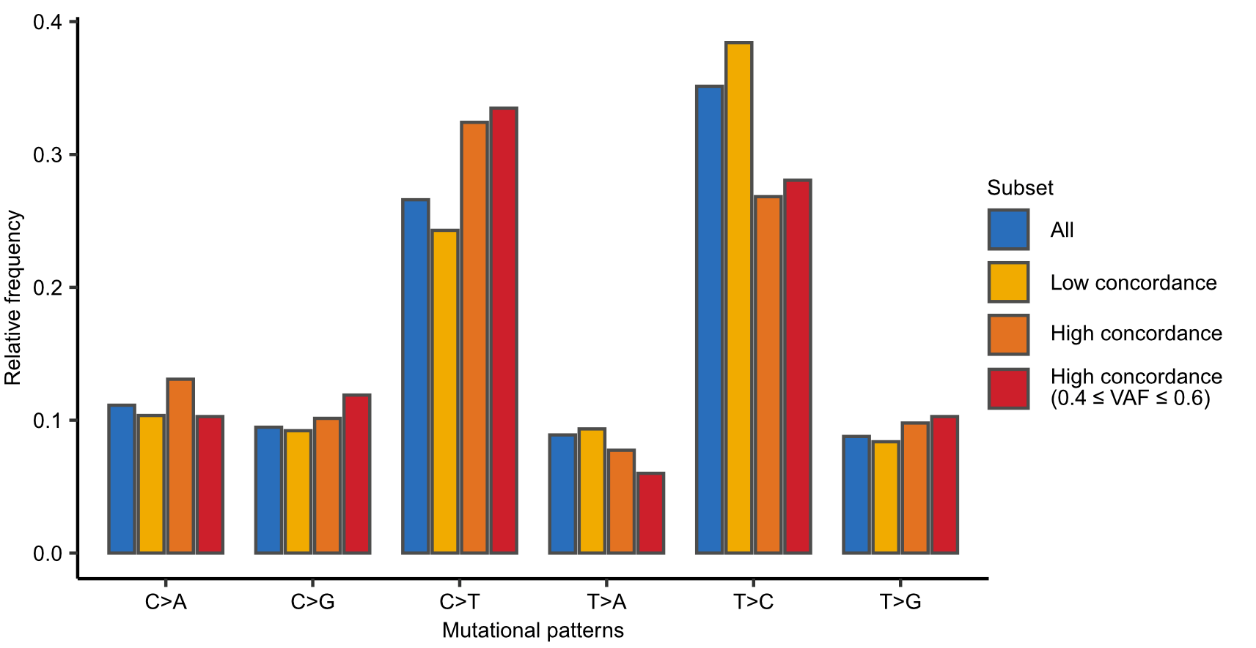


**Supplementary Fig. 4. Mutational patterns of postzygotic somatic mutations.**

The bar plots show the relative frequencies of six different substitution-type mutational patterns across the subsets. The subsets include the mutations from all MZ twins (All), twins with genotype concordance rate below 0.99 (Low concordance), twins with genotype concordance rate above 0.99 (High concordance), and mutations with VAFs around 0.5 in high concordant twins.


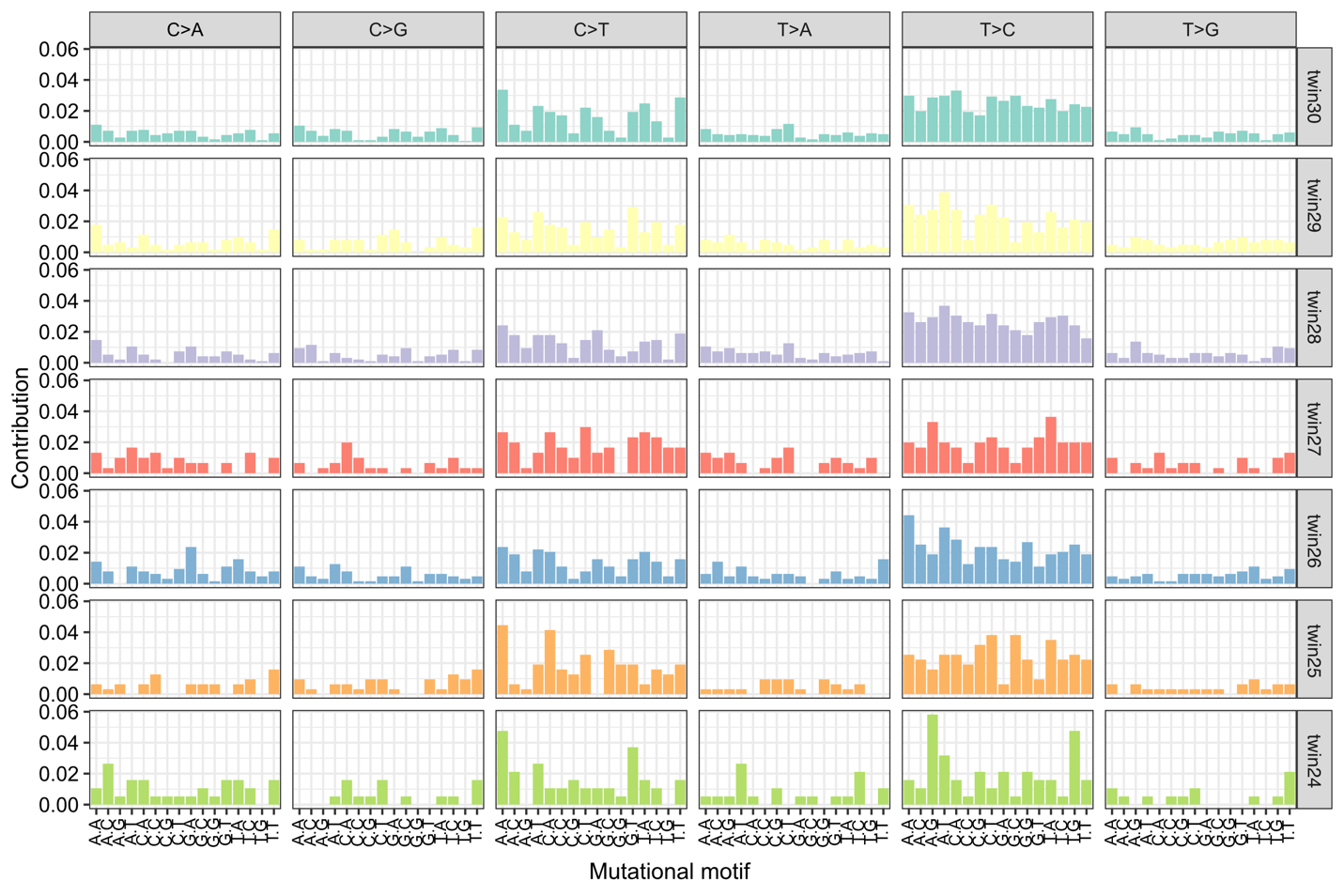


**Supplementary Fig. 5. The seven mutational signatures of MZ twins with low genotype concordance rate.**

The mutational signature patterns of postzygotic somatic mutations between MZ twins with the genotype concordance rate less than 99%. The columns represent the six base substitutions and rows are each twin pair. The x-axis indicates the mutational motif incorporating 3’ and 5’ bases with the substitution. The bar plot indicates the proportion of the mutational motif.

**Supplementary Table 1**. **The detailed information of several postzygotic somatic mutations between MZ twins targeted by Sanger sequencing.**

| **Chromosome** | **Position(hg38)** | **Reference/ Alternative** | **Variant allele frequency** | **Gene** | **Function** | **Validation (Sanger sequencing)** |
| --- | --- | --- | --- | --- | --- | --- |
| chr1 | 12293630 | G/T | 0.7 | VPS13D | Missense | No |
| chr1 | 145872601 | G/T | 0.2 | ANKRD35 | Missense | Yes |
| chr1 | 228406258 | A/G | 0.75 | TRIM11 | Missense | No |
| chr2 | 63404462 | T/C | 0.33 | WDPCP | Missense | No |
| chr3 | 44933160 | C/T | 0.2 | ZDHHC3 | Missense | No |
| chr4 | 78384139 | C/T | 0.39 | FRAS1 | Missense | No |
| chr6 | 42106410 | T/G | 0.5 | C6orf132 | Missense | No |
| chr7 | 44116965 | C/T | 0.18 | POLD2 | Missense | No |
| chr10 | 47349739 | G/A | 0.58 | RBP3 | Missense | No |
| chr11 | 110164992 | G/A | 0.52 | ZC3H12C | Missense | No |
| chr14 | 94587454 | T/C | 0.64 | SERPINA5 | Missense | No |
| chr16 | 3205168 | G/T | 0.41 | OR1F1 | Missense | No |
| chr19 | 45517699 | G/A | 0.44 | VASP | Missense | No |
| chr19 | 57606534 | A/G | 0.16 | ZNF530 | Missense | No |
| chr20 | 34260517 | C/T | 0.28 | ASIP | Missense | No |
| chr20 | 63559248 | A/G | 0.25 | HELZ2 | Stoploss | No |
